# Ecology and Epidemiology of Tickborne Pathogens, Washington, USA, 2011–2016

**DOI:** 10.3201/eid2604.191382

**Published:** 2020-04

**Authors:** Elizabeth A. Dykstra, Hanna N. Oltean, David Kangiser, Nicola Marsden-Haug, Stephen M. Rich, Guang Xu, Min-Kuang Lee, Muhammad G. Morshed, Christine B. Graham, Rebecca J. Eisen

**Affiliations:** Washington State Department of Health, Olympia, Washington, USA (E.A. Dykstra, H.N. Oltean, D. Kangiser, N. Marsden-Haug);; University of Massachusetts–Amherst, Amherst, Massachusetts, USA (S.M. Rich, G. Xu);; British Columbia Centre for Disease Control, Vancouver, British Columbia, Canada (M.-K. Lee, M.G. Morshed);; Centers for Disease Control and Prevention, Fort Collins, Colorado, USA (C.B. Graham, R.J. Eisen)

**Keywords:** Tickborne disease, *Ixodes*, *Dermacentor*, Washington, vector-borne infections, United States, ticks, epidemiology, ecology, zoonoses, bacteria, parasites

## Abstract

Tickborne diseases are rare in Washington, USA, and the ecology of these pathogens is poorly understood. We integrated surveillance data from humans and ticks to better describe their epidemiology and ecology. During 2011–2016, a total of 202 tickborne disease cases were reported in Washington residents. Of these, 68 (34%) were autochthonous, including cases of Lyme disease, Rocky Mountain spotted fever, tickborne relapsing fever, and tularemia. During May 2011–December 2016, we collected 977 host-seeking ticks, including *Ixodes pacificus*, *I. angustus*, *I. spinipalpis*, *I. auritulus*, *Dermacentor andersoni*, and *D. variabilis* ticks. The prevalence of *Borrelia burgdorferi* sensu stricto in *I. pacificus* ticks was 4.0%; of *B. burgdorferi* sensu lato, 3.8%; of *B. miyamotoi*, 4.4%; and of *Anaplasma phagocytophilum*, 1.9%. We did not detect *Rickettsia rickettsii* in either *Dermacentor* species. Case-patient histories and detection of pathogens in field-collected ticks indicate that several tickborne pathogens are endemic to Washington.

## Abstract 2

Vector populations in Washington, USA, are infected with several disease-causing pathogens.

Tickborne infections are the most common cause of vectorborne disease in the United States (*1*). Disease epidemiology is complex, the result of many causes, such as spatiotemporal variation in infected vectors, human behavior, reservoir host abundance, and climate variation (*1*,*2*). Compared with highly endemic areas such as the upper Midwest and northeastern United States, for the state of Washington, the epidemiology and ecology of tickborne diseases is not well characterized. Washington is divided into 39 counties spread across multiple, distinct ecosystems; the diverse ecology presents a range of potential vector habitats.

Human Lyme disease cases, with and without travel outside the state within the exposure period, have been confirmed in Washington (*3*). *Borrelia burgdorferi* sensu stricto (s.s.)–infected *Ixodes pacificus* ticks have been documented in far western North America, including California, USA, and British Columbia, Canada (*4*–*6*). In addition, a recent study in Washington documented canine seroprevalence of 3.8 cases/1,000 dogs (*7*). *Anaplasma phagocytophilum* has been reported in small mammals (*8*,*9*) and in vector ticks in California (*10*); clinical cases of anaplasmosis have been reported in dogs from California to British Columbia (*7*,*11*,*12*).

Rare cases of autochthonous babesiosis have been reported in Washington, 3 caused by *Babesia duncani* and 1 caused by a *B. divergens*–like organism (*13*–*15*). Evidence of *D. albipictus* ticks as the vector for *B. duncani* has only recently emerged (*16*).

Three of 11 *Ixodes* tick species (*I. pacificus*, *I. angustus*, and *I. spinipalpis*) reported from Washington are known or suspected vectors for tickborne diseases (*17*). *I. pacificus* ticks are frequent human-biters, and the species is an established vector of *B. burgdorferi* s.s. and *A. phagocytophilum* and a putative vector of *B. miyamotoi* (*18*). *I. angustus* ticks can experimentally transmit *B. burgdorferi* s.s. and might play a role in the spirochete’s enzootic cycle (*19*,*20*); likewise, *I. spinipalpis* ticks might play a role in the natural maintenance of *B. burgdorferi* sensu lato (s.l.) (*21*).

Competent reservoirs for *B. burgdorferi* s.s*.*, including deer mice (*Peromyscus maniculatus*), western gray squirrels (*Sciurus griseus*), and several *Tamias* spp. chipmunks, are found in Washington (*22*–*25*). *P. maniculatus* deer mice have been found infected with *B. burgdorferi* s.l. in western Washington (*22*). Although not recognized as human pathogen reservoirs, lizards are notable blood-meal hosts for immature *I. pacificus* ticks (*26*,*27*) and 3 lizard species are found in Washington: northern alligator lizard (*Elgaria coerulea*), southern alligator lizard (*E. multicarinata*), and the western fence lizard (*Sceloporus occidentalis*) (*25*,*28*; C.S. Arnason, Biology of the western black-legged tick, *Ixodes pacificus*, (Cooley and Kohls, 1943): a potential vector of Lyme disease in south coastal British Columbia [master’s thesis], Vancouver: Simon Fraser University; 1992). Both *E. multicarinata* and *S. occidentalis* lizards are zooprophylactic against *B. burgdorferi*.

Autochthonous cases of Rocky Mountain spotted fever (RMSF) were reported in Washington each year until the 1940s (*29*). To date, there is no published evidence of *R. rickettsii* in ticks collected in Washington. Tularemia is prevalent throughout the Northern Hemisphere and occurs in many animal species (*30*). Recent *Francisella tularensis* antibody detections were reported from wildlife in Idaho (*31*). Up to 10 cases of tularemia are reported each year in Washington (*29*). *D. andersoni* and *D. variabilis* ticks, both competent vectors of *R. rickettsii* and *F. tularensis*, occur in the state (*18*,*32*). The brown dog tick, *Rhipicephalus sanguineus*, a known vector of RMSF in the southwest, is also reportedly present (*33*,*34*).

*Borrelia hermsii*, the causative agent of tickborne relapsing fever (TBRF), occurs in Washington and is vectored by *Ornithodoros hermsi*, a soft tick (family Argasidae) typically found in rodent nests (*35*,*36*). TBRF is the most commonly reported autochthonous tickborne disease in Washington; up to 12 cases are reported annually (*29*). The first documented evidence of canine infection with *B. hermsii* was reported in a dog with travel to Chelan County, Washington (*37*). *B. hermsii*–positive *O. hermsi* ticks have also been documented in Washington (*38*).

Human cases of Lyme disease, anaplasmosis, ehrlichiosis, babesiosis, spotted fever rickettsioses (including RMSF), TBRF, and tularemia are reportable to local health jurisdictions in Washington. However, clinical underrecognition and underreporting of disease are suspected. To clarify the epidemiology of tickborne diseases in Washington, we analyzed locally acquired cases and tick surveillance data. Our objectives were to describe tickborne disease epidemiology among autochthonous human cases in Washington during the study period, as well as Ixodid vectors and pathogen detections in ticks collected in Washington.

## Materials and Methods

### Human Case Identification

Human tickborne disease cases are identified through mandatory, but passive, reporting to local health jurisdictions from Washington healthcare providers and laboratories testing Washington residents. We reviewed all cases of anaplasmosis, ehrlichiosis, Lyme disease, babesiosis, TBRF, RMSF, and tularemia reported during 2011–2016. To ensure comparability over time, we reclassified cases to the Council for State and Territorial Epidemiologists case definitions as of 2017. Confirmed and probable cases were included for each condition. Reclassifications were required for Lyme disease, babesiosis, and tularemia. Local health jurisdictions interviewed cases in the year of report to determine clinical course, travel history, and most likely exposure location. Cases were classified as locally acquired (in-state), out-of-state acquired, or unknown exposure location based on a standardized definition. We evaluated frequency distribution of demographic variables for each condition with locally acquired cases.

### Tick Surveillance

Washington State Department of Health (DOH) staff conducted weekly or biweekly tick drags during March–October at 15 sites in 5 counties in western Washington that were identified as having suitable tick habitat, public access, and relative proximity to DOH offices, thus allowing frequent monitoring. We sampled 7 sites regularly for >2 years and 8 sites for 1 year. Sampling was also performed in 2 counties deemed most likely exposure locations for locally acquired Lyme disease cases reported during 2011–2016. We conducted surveillance using tick drags, the most effective sampling method for both *Ixodes* and *Dermacentor* ticks. Lack of resources and capacity prevented us from including Argasid tick surveillance as part of this study. We sampled by dragging a 1 m^2^ piece of flannel on the ground along either a 30-m transect or for 30 minutes in a plot created in a specific vegetation type. We inspected drags for ticks every 3–6 meters. We also obtained ticks from partners in 15 counties who found unattached, unfed ticks on themselves and reported GPS collection locations.

Upon collection, we speciated ticks using standard taxonomic keys, then stored them in vials of 95% ethanol at 4°C (*17*,*39*,*40*). We submitted specimens to either the Laboratory of Medical Zoology, University of Massachusetts–Amherst (Amherst, MA, USA); the Centre for Disease Control, British Columbia (Vancouver, BC, Canada); or the US Centers for Disease Control and Prevention (Fort Collins, CO, USA) for pathogen testing.

### DNA Extraction and Molecular Identification

Pathogen testing varied by laboratory and over time; groups of ticks were tested by different laboratories for different pathogens. Testing of *Ixodes* and *Dermacentor* ticks followed each laboratory’s protocols (*33*,*41*–*43*). *Ixodes* ticks were tested for *A. phagocytophilum*, *B. burgdorferi* s.s. and s.l., *B. miyamotoi*, *B. mayonii*, *Babesia* spp., *B. microti*, *Ehrlichia muris*–like agent, Powassan virus, Heartland virus, Colorado tick fever virus, and Bourbon virus. *Dermacentor* ticks were tested for *F. tularensis*, *R. rickettsii*, Powassan virus, Heartland virus, Colorado tick fever virus, and Bourbon virus. *B. burgdorferi* s.l. detected in ticks tested before 2015 were not subspeciated.

## Results

**Figure 1:**
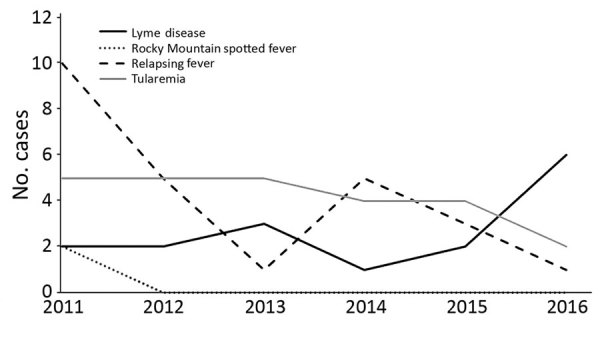
Locally acquired cases of tickborne diseases, Washington, USA, 2011–2016.

**Figure 2:**
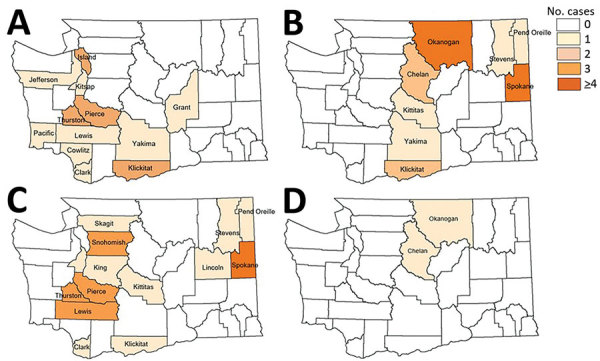
Counties of likely exposure for autochthonous human tickborne disease cases, Washington, USA, 2011–2016. A) Lyme disease; B) tickborne relapsing fever; C) tularemia; D) Rocky Mountain spotted fever.

During 2011–2016, a total of 202 cases of tickborne disease were reported in Washington residents; because of reclassification, this number does not match what is reported in Centers for Disease Control and Prevention notifiable condition data. Of these cases, 68 (34%) were autochthonous: Lyme disease (16 cases), RMSF (2 cases), TBRF (25 cases), and tularemia (25 cases). Yearly counts of locally acquired tickborne disease cases were low; <20 cases were reported annually (Figure 1). Tularemia and TBRF were the most frequently reported autochthonous tickborne diseases. All TBRF exposures were in eastern Washington, most in Okanogan and Spokane counties, whereas tularemia cases were broadly distributed (Figure 2). Low numbers (2–6 cases) of locally acquired Lyme disease were reported each year; for each case, no travel outside Washington during exposure periods was reported. We determined likely exposure locations based on exposure to tick habitat or known tick bite if travel to multiple counties occurred during the exposure period; these cases involved 12 counties in both eastern and western Washington. 

Only 2 probable cases of RMSF were reported; both met the minimum IgG detection value. One case-patient experienced fever, lymphadenopathy, and a single ulcerated lesion. The second case-patient reported fever and myalgia with no rash and reported a known tick bite; however, the tick was detected after symptom onset. Both patients reported likely exposure in north central Washington. 

Tickborne diseases cases were reported throughout the year; the highest case counts occurred during April–October. Lyme disease cases in May, tularemia cases in July, and TBRF cases in September.

**Figure 3:**
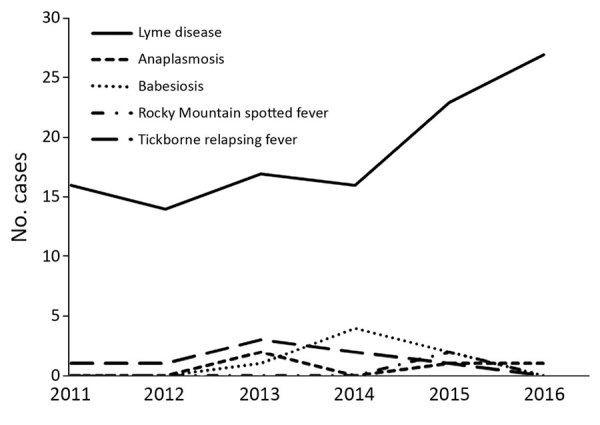
Travel-associated cases of tickborne diseases, Washington, USA, 2011–2016.

Lyme disease was the most commonly reported imported tickborne disease, and overall case counts of imported Lyme disease increased over the study period (Figure 3). Low numbers of travel-associated anaplasmosis, babesiosis, RMSF, and TBRF were reported. Two cases of blood transfusion–associated babesiosis were reported, 1 in 2014 and 1 in 2015. The blood donors in each case were Washington residents with travel history to babesiosis-endemic states (Massachusetts and Connecticut). No human cases of *B. miyamotoi* infection were reported in Washington residents during this period.

We identified no statistically significant differences in age or gender distribution between case-patients with locally acquired tickborne disease and those with imported cases or unknown exposure history. Among autochthonous cases, 43% of patients were female and 57% male; patient ages ranged from 7 to 91 years (median 49 years). Patients with imported cases were 39% female and 61% male; ages in this group ranged from 3 to 87 years (median 49 years).

During 2011–2016, we collected 977 unfed, host-seeking ticks from 53 sites in 19 counties (Appendix Tables 1, 2): *I. pacificus* (n = 438), *I. spinipalpis* (n = 236), *I. angustus* (n = 99), *I. auritulus* (n = 5), *D. andersoni* (n = 151), and *D. variabilis* (n = 46). Two *Ixodes* larvae were unspeciated. The 3 primary vector species, *I. pacificus*, *D. andersoni*, and *D. variabilis*, were active predominantly during the spring; 576/635 (91%) ticks were collected during March–May. Most ticks collected were adults: 100% *D. andersoni* and *D. variabilis* and 96% (420/438) *I. pacificus*.

**Figure 4:**
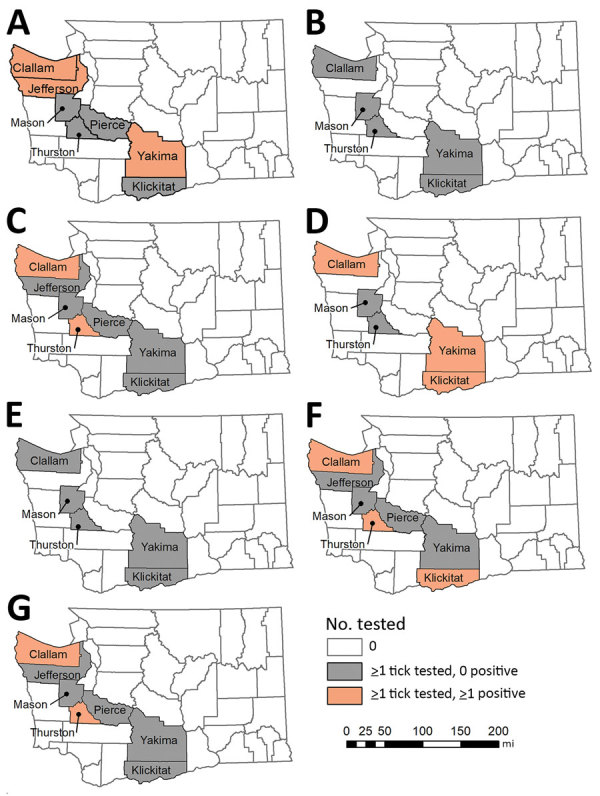
Counties with collection and testing of unfed, field-collected *Ixodes pacificus* ticks and pathogen detections, Washington, USA, 2011–2016. A) *Anaplasma phagocytophilum*; B) *Borrelia bissettiae*; C) *B. burgdorferi* sensu lato; D) *B. burgdorferi* sensu stricto; E) *B. lanei*; F) *B. miyamotoi*; G) *Borrelia* species.

We detected *B. burgdorferi* s.s. in 14/354 (4.0%) *I. pacificus* ticks (Table). However, detections were from only 3 of 5 counties where *B. burgdorferi* subspeciation was conducted: Clallam, 11/121 (9.1%); Klickitat, 2/117 (1.7%); and Yakima, 1/3 (33.3%) (Figure 4). In addition, we detected *B. burgdorferi* s.l. in 16/421 (3.8%) and *B. miyamotoi* in 10/227 (4.4%) *I. pacificus* ticks and *A. phagocytophilum* in 5/258 (1.9%) *I. pacificus* ticks. Six *I. pacificus* ticks were co-infected with 2 pathogens: 4 with *B. burgdorferi* s.s. and *B. miyamotoi*, 1 with *B. burgdorferi* s.s. and *A. phagocytophilum*, and 1 with *Borrelia* spp. and *A. phagocytophilum*. We also found *A. phagocytophilum* in 1/234 (0.4%) *I. spinipalpis* ticks. We detected *B. burgdorferi* s.l. in 4/235 (1.7%) *I. spinipalpis* ticks and in 1/99 (1.0%) *I. angustus* ticks. We did not detect *R. rickettsii* or *F. tularensis* in any field-collected *Dermacentor* ticks.

**Table:** Pathogens detected in unfed, field-collected *Ixodes* species ticks, Washington state, 2011–2016

Pathogen	No. positive/no. tested (%)									
	*I. angustus*			*I. pacificus*				*I. spinipalpis*		
	Adult	Nymphs		Adult	Nymphs	Larvae		Adults	Nymphs	Larvae
*Anaplasma phagocytophilum*	0/79	0/16		5/240 (2.1)	0/17	0/1		1/4 (25.0)	0/122	0/108
*Borrelia* species*	1/82 (1.2)	0/16		4/361 (1.1)	0/17	0/1		1/5 (20.0)	1/122 (0.8)	0/108
*Borrelia burgdorferi* sensu lato	1/83 (1.2)	0/16		22/403 (5.5)	0/17	0/1		1/5 (20.0)	3/122 (4.1)	0/108
*B. burgdorferi* sensu stricto	0/41	0/4		14/340 (4.1)	1/14 (7.1)	0/0		0/3	0/63	0/0
*B. miyamotoi*	0/38	0/4		10/211 (4.7)	0/16	0/0		0/2	0/67	0/0

*Not differentiated to species.

## Discussion

Although Washington is considered a low-incidence state for tickborne diseases, our results indicate that vector populations in this state are infected with several disease-causing agents. Tickborne pathogens now considered endemic in at least some areas of Washington include *B. burgdorferi* s.s., *B. hermsii*, *B. miyamotoi*, and *F. tularensis.* Evidence exists for the presence of *A. phagocytophilum*, *B. duncani*, and *R. rickettsii*, but further information is needed to assess risk. Human and animal healthcare providers should be aware of the possible risk of these diseases in their patients and should be vigilant for consistent symptoms paired with exposure histories.

Tick and human surveillance led to a common picture of increased activity in the spring. *I. pacificus* ticks infected with *B. burgdorferi* s.s., *B. miyamotoi*, or *A. phagocytophilum* were found in 4 of 9 surveyed counties. Human Lyme disease case-patients reported exposures in 12 counties. However, overlap in infected vector populations and human cases of Lyme disease occurred in just 2 counties, Klickitat and Yakima. In the remaining 10 counties, we found no positive detections of *B. burgdorferi* s.s. Field surveillance was not performed in 7 of these counties (Clark, Cowlitz, Grant, Island, Kitsap, Lewis, and Pacific); in 2 (Jefferson, Pierce), all field-collected *I. pacificus* tested negative or were not tested; and in 1 (Thurston), *B. burgdorferi* s.l. was detected, but not subspeciated. No autochthonous human cases of anaplasmosis were reported, so there was no overlap with infected vector populations. Lack of systematic tick sampling in several of these counties, owing to their distance from DOH and resource capacity restraints, resulted in few or no unfed ticks collected, thereby limiting pathogen detection. Additional surveillance in these areas is needed to better describe the pathogen prevalence and potential for human–tick encounters.

The higher prevalence of *B. burgdorferi* s.l. (8.8%) and *B. burgdorferi* s.s. (9.1%) in *I. pacificus* ticks found in Clallam County suggests that >1 competent reservoir host exists in the area. The only zooprophylactic host in the area, the northern alligator lizard (*E. coerulea*), is uncommon at the sites where these ticks were collected, which might be a contributing factor to the higher pathogen prevalence. Alternatively, the small area sampled might be producing unstable prevalence estimates; additional sampling is needed to increase confidence in these findings. No human case-patients with Lyme disease reported exposure in Clallam County, possibly because of limited human–tick interaction in this area; further studies are needed to determine the most likely reservoir and to better describe human–vector interactions. All dually infected *I. pacificus* ticks also were collected in Clallam County.

In contrast to Clallam County, Klickitat County *I. pacificus* ticks had much lower prevalence of *B. burgdorferi* s.s., but 2 human patients with Lyme disease reported exposure there. The hotter, drier habitat of Klickitat County supports populations of all 3 Washington lizard species, which could be a contributing factor to why, despite the abundant tick population, the pathogen prevalence is lower.

The almost total lack of pathogen detection in field-collected *I. angustus* ticks suggests that this species plays little or no role in the maintenance or transmission of *B. burgdorferi* in Washington. This finding is confirmed by reports from California, Oregon, and Washington (*44*). Small numbers of both *I. spinipalpis* and *I. angustus* ticks have been found attached to humans in Washington and submitted to DOH for identification, but their role in pathogen transmission remains unknown.

*B. miyamotoi* was detected at a similar prevalence in *I. pacificus* adults as *B. burgdorferi* s.s., which is contrary to what has been found in other states, where prevalence of *B. burgdorferi* s.s. is often 10-fold higher than *B. miyamotoi* (*45*). No human cases of *B. miyamotoi* disease have been reported in Washington, which is likely attributable to a lack of clinical suspicion and testing but could also be attributable to *I. pacificus* ticks being a less efficient vector of *B. miyamotoi* than of *B. burgdorferi* s.s.

*A. phagocytophilum* has been reported from dogs, but not humans, in Washington. Strain variation of *A. phagocytophilum* with specific host tropism has been described (*46*–*48*); it is unknown whether the strain in Washington is not pathogenic to humans or whether the lack of detection in humans is the result of clinical underrecognition. *I. pacificus* ticks appear to play a primary role in maintaining this pathogen in nature, although *I. spinipalpis* ticks might play a minor role.

We detected no *Babesia* species in any of the ticks tested. A recent study implicating *D. albipictus* ticks as the probable vector of *B. duncani* suggests that the appropriate tick species was not tested.

Further, we found no detections of *R. rickettsii* or *F. tularensis* in unfed ticks, which is consistent with findings in other states and suggests that both these pathogens are very rare in vector populations. The presence of 2 nonpathogenic strains of *Rickettsia*, including *R. peacocki*, which is refractory to infection with and maintenance of *R. rickettsii*, suggests that *R. rickettsii* could be present only in focal areas, which is consistent with other findings (*49*). Very low or zero prevalence of *R. rickettsii* is supported by human case data; only 2 probable cases were reported during the study period. Whereas tularemia is relatively common, the transmission routes for *F. tularensis* are varied and not limited to tick vectors (*50*).

Several limitations exist with our study. Field surveillance was conducted at a small number of sites because of limited resources and efforts to determine temporal tick activity. This resulted in inconsistent and largely convenience-based tick surveillance coverage across the state. There remains a paucity of understanding of what specific reservoirs drive the maintenance of these pathogens in nature. However, several known, competent reservoirs for *B. burgdorferi* s.s. exist in counties where pathogens were detected in the tick population. Little is currently known about the epidemiology of *R. rickettsii* in Washington.

All human case reports described here arose from passive surveillance systems; locally acquired cases required positive laboratory results. Underdiagnosis and underreporting of tickborne disease are likely, as patients might not seek healthcare and healthcare providers might be unaware of the possibility. In addition, common laboratory tests might be negative early in the course of illness and true cases could be missed, particularly if serologic testing is ordered early, rather than nucleic acid detection tests. In contrast, many of the diagnostic tests used for tickborne diseases have poor specificity (e.g., Lyme disease antibody testing) and might cross-react with other species (e.g., *Rickettsia* testing). The application of these tests in a low-incidence setting decreases their positive predictive value, and some of the cases included in this analysis likely represent false-positive results. 

The same is likely true for many of the probable Lyme disease cases for which symptoms did not meet the clinical criteria set in the Council for State and Territorial Epidemiologists case definition or for which symptom information was not available. Detections of Lyme disease in 4 counties (Grant, Jefferson, Kitsap, and Lewis) were based on a single probable case each; additional evidence for *B. burgdorferi* s.s. in ticks in these counties would help lend certainty to these findings. With the exception of Grant County, submissions from veterinarians and the general public indicate that *I. pacificus* ticks are present in those counties. Similarly, both reported RMSF cases met the minimum cutoff value for IgG; based on clinical histories, these results were likely false-positive. Some misclassification of human case exposure location is probable because there is no way to determine exposure location with certainty. Finally, unknown tickborne disease pathogens could be present in Washington for which diagnostic tests are not available. As awareness of tickborne diseases spreads in the general population and among healthcare providers, we could see an increase in the number of cases as a result of improvements in diagnosis and reporting.

Strengths of this study include tracking tick collection methods and feeding status, which enabled stratification of tick data for analysis of only field-collected, unfed ticks. Submissions from host-collected ticks might not represent the true distribution in Washington, instead reflecting the host’s travel history and potentially distorting estimates of prevalence. In addition, field surveillance drags were conducted at known sites and, in most cases, at multiple times during the year, providing a better picture of seasonal tick activity. Testing of individual ticks, as opposed to pooling, provided more exact information about pathogen prevalence in each site’s tick population and allowed us to assess co-infection rates in individual ticks. All but a very few ticks were identified to species before testing.

We interviewed all human case-patients for exposure history, including travel, enabling us to distinguish travel-related cases from possible autochthonous cases, which is crucial to understanding tickborne disease burden in Washington. The analysis of human and tick data in tandem allowed for a more comprehensive picture of pathogen distributions and prevalence in Washington than analyzing either alone.

The true underlying rate of tickborne diseases in Washington remains unknown. Several human and animal pathogens found in tick populations are endemic to Washington, including *B. burgdorferi* s.s., *Babesia* spp., *F. tularensis*, *B. hermsii*, *A. phagocytophilum*, *B. miyamotoi*, and *R. rickettsii*; healthcare providers should be vigilant for symptoms of disease and exposure histories. The rarity of tickborne diseases creates a surveillance and diagnostic challenge; it is difficult to maintain awareness and clinical suspicion for these conditions in low-incidence settings. Surveillance data from field-collected ticks identified areas of potential human risk unidentified by existing human surveillance. Ongoing surveillance of both human cases and tick vectors is required to determine the true burden of disease and to improve public health prevention messaging to healthcare providers and the public.

Appendix Additional information on the epidemiology of tickborne pathogens, Washington, 2011–2016.
