# Supplementary material for: Ecology and Epidemiology of Tickborne Pathogens, Washington, USA, 2011–2016
Source: Emerg Infect Dis. 2020 Apr;26(4):648–57. doi: 10.3201/eid2604.191382 (PMC7101130; doi:10.3201/eid2604.191382)
Supplement: Appendix — Additional information on the epidemiology of tickborne pathogens, Washington, 2011–2016. [file 19-1382-Techapp-s1.pdf]

Article DOI: https://doi.org/10.3201/eid2604.191382

Ecology and Epidemiology of Tickborne Pathogens, Washington, USA, 2011–2016

Appendix

Appendix Table 1. Pathogens detected in unfed, field-collected, adult *Ixodes* ticks by county, Washington, 2011–2016

Pathogen, county	No. positive/no. tested (%)									
	I. angustus		*I. auritulus*		*I. pacificus*			*I. spinipalpis*		
	**Adults**	Nymphs	Adults	Nymphs	Adults	Nymphs	Larvae	Adults	Nymphs	Larvae
Anaplasma phagocytophilum, total	0/79	0/16	0/1	0/4	5/240 (2.1)	0/17	0/1	1/4 (25.0)	0/122	0/108
Clallam	0/22	0/2	0/0	0/4	4/88 (4.8)	0/6	0/0	0/1	0/36	0/0
Jefferson	0/0	0/0	0/0	0/0	0/1	0/0	0/0	0/0	0/0	0/0
Mason	0/9	0/4	0/0	0/0	0/1	0/4	0/0	1/3 (33.3)	0/58	0/0
King	0/9	0/2	0/0	0/0	0/0	0/0	0/0	0/0	0/0	0/0
Klickitat	0/0	0	0/0	0/0	0/51	0/4	0/0	0/0	0/0	0/0
Pierce	0/17	0/2	0/0	0/0	0/2	0/0	0/1	0/0	0/7	0/108
Pacific	0/1	0	0/0	0/0	0/0	0/0	0/0	0/0	0/0	0/0
Thurston	0/21	0/6	0/1	0/0	0/94	0/3	0/0	0/0	0/21	0/0
Yakima	0/0	0	0/0	0/0	1/3 (33.3)	0/0	0/0	0/0	0/0	0/0
Borrelia burgdorferi sensu stricto, total	0/41	0/4	0/0	0/4	14/340 (4.1)	1/14 (7.1)	0/0	0/3	0/63	0/0
Clallam	0/22	0/2	0/0	0/4	12/115 (10.4)	0/6	0/0	0/1	0/36	0/0
Klickitat	0/0	0/0	0/0	0/0	1/113 (0.9)	1/4 (25.0)	0/0	0/0	0/0	0/0
Mason	0/4	0/0	0/0	0/0	0/4	0/2	0/0	0/2	0/8	0/0
Pacific	0/1	0/0	0/0	0/0	0/0	0/0	0/0	0/0	0/0	0/0
Pierce	0/1	0/0	0/0	0/0	0/0	0/0	0/0	0/0	0/0	0/0
Thurston	0/11	0/2	0/0	0/0	0/105	0/2	0/0	0/0	0/19	0/0
Yakima	0/0	0/0	0/0	0/0	1/3 (33.3)	0/0	0/0	0/0	0/0	0/0
Borrelia burgdorferi sensu lato, total	1/99 (1.0)	0/14	0/1	0/4	16/403 (4.0)	0/17	0/1	1/5 (20.0)	3/122 (2.5)	0/108
Clallam	0/22	0/2	0/0	0/4	7/74 (9.5)	0/6	0/0	0/1	0/36	0/0
Jefferson	0/0	0/0	0/0	0/0	0/1	0/0	0/0	0/0	0/0	0/0
Klickitat	0/0	0/0	0/0	0/0	1/50 (2.0)	0/4	0/0	0/0	0/0	0/0
Mason	0/4	0/0	0/0	0/0	0/1	0/3	0/0	0/1	0/11	0/0
Pacific	0/1	0/0	0/0	0/0	0/0	0/0	0/0	0/0	0/0	0/0
Pierce	0/1	0/0	0/0	0/0	0/1	0/0	0/0	0/0	0/0	0/0
Thurston	0/10	0/2	0/0	0/0	2/81 (2.5)	0/3	0/0	0/0	0/20	0/0
Yakima	0/0	0/0	0/0	0/0	0/3	0/0	0/0	0/0	0/0	0/0
Borrelia miyamotoi, total	0/38	0/4	0/0	0/4	10/211 (4.7)	0/16	0/0	0/2	0/67	0/0
Clallam	0/22	0/2	0/0	0/4	7/74 (9.5)	0/6	0/0	0/1	0/36	0/0
Jefferson	0/0	0/0	0/0	0/0	0/1	0/0	0/0	0/0	0/0	0/0
Klickitat	0/0	0/0	0/0	0/0	1/50 (2.0)	0/4	0/0	0/0	0/0	0/0
Mason	0/4	0/0	0/0	0/0	0/1	0/3	0/0	0/1	0/11	0/0
Pacific	0/1	0/0	0/0	0/0	0/0	0/0	0/0	0/0	0/0	0/0
Pierce	0/1	0/0	0/0	0/0	0/1	0/0	0/0	0/0	0/0	0/0
Thurston	0/10	0/2	0/0	0/0	2/81 (2.5)	0/3	0/0	0/0	0/20	0/0
Yakima	0/0	0/0	0/0	0/0	0/3	0/0	0/0	0/0	0/0	0/0
Borrelia mayonii, total	1/99 (1.0)	0/14	0/1	0/4	16/403 (4.0)	0/17	0/1	1/5 (20.0)	3/122 (2.5)	0/108
Clallam	0/22	0/2	0/0	0/4	0/59	0/6	0/0	0/1	0/35	0/0
Klickitat	0/0	0/0	0/0	0/0	0/50	0/4	0/0	0/0	0/0	0/0
Mason	0/4	0/0	0/0	0/0	0/1	0/2	0/0	0/1	0/8	0/0
Pacific	0/1	0/0	0/0	0/0	0/0	0/0	0/0	0/0	0/0	0/0
Pierce	0/1	0/0	0/0	0/0	0/0	0/0	0/0	0/0	0/0	0/0
Thurston	0/9	0/2	0/0	0/0	0/75	0/2	0/0	0/0	0/19	0/0

Pathogen, county	*I. angustus*		*I. auritulus*		*I. pacificus*			*I. spinipalpis*		
	No. positive/no. tested (%)									
	**Adults**	Nymphs	Adults	Nymphs	Adults	Nymphs	Larvae	Adults	Nymphs	Larvae
Yakima	0/0	0/0	0/0	0/0	0/2	0/0	0/0	0/0	0/0	0/0
Borrelia species, total	1/82 (1.2)	0/16	0/1	1/4 (25.0)	4/361 (1.1)	0/17	0/1	1/5 (20.0)	1/122 (0.8)	0/108
Clallam	0/24	0/2	0/0	1/4 (25.0)	3/133 (2.3)	0/6	0/0	0/1	0/37	0/0
Jefferson	0/0	0/0	0/0	0/0	0/1	0/0	0/0	0/0	0/0	0/0
King	0/9	0/2	0/0	0/0	0/0	0/0	0/0	0/0	0/0	0/0
Klickitat	0/0	0/0	0/0	0/0	0/111	0/4	0/0	0/0	0/0	0/0
Mason	1/9 (11.1)	0/4	0/0	0/0	0/4	0/4	0/0	1/4 (25.0)	1/58 (1.7)	0/0
Pacific	0/1	0/0	0/0	0/0	0/0	0/0	0/0	0/0	0/0	0/0
Pierce	0/17	0/2	0/0	0/0	0/2	0/0	0/1	0/0	0/7	0/108
Thurston	0/22	0/6	0/1	0/0	1/107 (0.9)	0/3	0/0	0/0	0/21	0/0
Yakima	0/0	0/0	0/0	0/0	0/3	0/0	0/0	0/0	0/0	0/0

Appendix Table 2. Pathogens detected in unfed field-collected, adult *Dermacentor* ticks, Washington, 2012–2016*

Pathogen	County	No. positive./no. tested (%) *D. andersoni*	*D. variabilis*
Rickettsia species, total		0/26	2/46 (4.3)
	Asotin	0/1	0/0
	Benton	0/0	0/3
	Chelan	0/1	0/0
	Ferry	0/1	0/0
	Franklin	0/0	0/1
	Grant	0/0	0/15
	King	0/0	0/1
	Kittitas	0/1	0/0
	Klickitat	0/1	1/12 (8.3)
	Lincoln	0/15	0/3
	Skamania	0/0	0/1
	Spokane	0/5	1/8 (12.5)
	Yakima	0/0	0/2
Rickettsia peacocki, total		8/22 (36)	2/42 (4.8)
	Benton	0/0	0/3
	Ferry	0/1	0/0
	Franklin	0/0	0/1
	Grant	0/0	1/15 (6.7)
	Kittitas	1/1 (100.0)	0/0
	Klickitat	0/0	0/11
	Lincoln	6/15 (40.0)	0/3
	Skamania	0/0	0/1
	Spokane	1/5 (20.0)	1/8 (12.5)
Rickettsia rhipicephali, total		2/22 (9.0)	1/42 (2.4)
	Benton	0/0	0/3
	Ferry	0/1	0/0
	Franklin	0/0	0/1
	Grant	0/0	0/15
	Kittitas	0/1	0/0
	Klickitat	0/0	1/11 (9.1)
	Lincoln	2/15 (13.3)	0/3
	Skamania	0/0	0/1
	Spokane	0/5	0/8

**Dermacentor* ticks were not actively collected until 2012.
